# Primary tubercular liver abscess in an immunocompetent adult: a case report

**DOI:** 10.1186/1752-1947-3-78

**Published:** 2009-10-15

**Authors:** CP Baveja, Vidyanidhi Gumma, Monica Chaudhary, Himanshu Jha

**Affiliations:** 1 Department of Microbiology, Tuberculosis Laboratory, Maulana Azad Medical College, New Delhi, India

## Abstract

**Introduction:**

Isolated primary tubercular abscess is one of the rare forms of extrapulmonary tuberculosis. A greater awareness of this rare clinical entity may help in commencing specific evidence-based therapy quickly and preventing undue morbidity and mortality.

**Case presentation:**

A 30-year-old man, of Asian origin, developed a hepatic tubercular abscess which was not associated with any pulmonary or gastrointestinal tract foci of tuberculosis. An ultrasonogram of the abdomen showed an abscess in the right lobe of his liver which was initially diagnosed as an amoebic liver abscess. Subsequently, the pus from the lesion yielded *Mycobacterium tuberculosis *using the BACTEC TB 460 instrument and *Mycobacterium tuberculosis *deoxyribonucleic acid by polymerase chain reaction. The patient was started on systemic antitubercular therapy to which he responded favorably.

**Conclusion:**

This report emphasizes the fact that, although a tuberculous liver abscess is a very rare entity, it should be included in the differential diagnosis of unknown hepatic mass lesions.

## Introduction

Though hepatic tuberculosis is not a rare disease entity, tubercular liver abscess (TLA) is extremely rare even in a country where tuberculosis is an alarming public health problem. It is usually associated with foci of infection either in the lung and/or gastrointestinal (GI) tract [1], or with an immunocompromised state. An isolated or primary TLA with no evidence of tuberculosis elsewhere is even rarer. The diagnosis is difficult in most instances and is frequently confused with hepatoma, pyogenic liver abscess or amoebic liver abscess [2-4]. The rarity of this clinical entity prompted us to present this case which involves an immunocompetent adult with an isolated hepatic tubercular abscess and with no foci of infection in the lungs or GI tract.

## Case presentation

A 30-year-old man of Asian origin, presented to the surgery outpatient department (OPD) with non-radiating pain in the right hypochondrium and epigastrium associated with vomiting, intermittent fever with chills and rigors for 10 days. The onset was gradual with increasing weakness and deterioration of his general health. The patient was undergoing follow-up for a liver abscess (right lobe) which was diagnosed previously as an amoebic liver abscess (ALA) based on a positive amoebic serology report from a private laboratory. He was treated conservatively for this and discharged 3 months before this present episode. There was no previous history of tuberculosis (TB) or contact with any patient with TB. At the time of admission, the patient was febrile, dehydrated and looked very ill and anemic with a pulse rate of 92/minute, blood pressure 106/70 mmHg and respiratory rate of 16/minute. There was no icterus and no lymphadenopathy. Abdominal examination revealed tenderness and guarding over the intercostal spaces overlying the liver. The liver span was 16 cm. There was no splenomegaly, ascites or any other palpable mass in his abdomen. Respiratory and cardiovascular system (CVS) examination revealed no abnormality.

**Figure 1:**
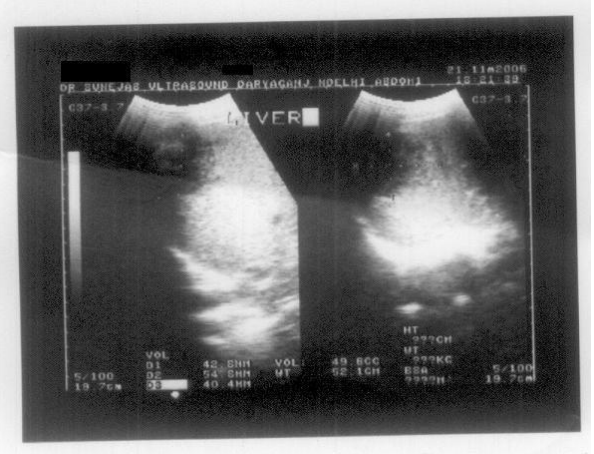
**Ultrasonogram of the liver showing a hypo-echoic lesion taken when the patient presented to the outpatient department for the first time**.

Chest X-ray showed no lesion suggestive of TB but revealed a right-sided subdiaphragmatic pathology as the right hemi-diaphragm was raised and the costophrenic angle was blunted. An ultrasonogram (USG) of the abdomen on the same day revealed a 5.6 × 6.8 × 8.8 cm ill-defined, heterogeneous hypo-echoic lesion reaching up to the liver surface with cystic areas in the right lobe of the liver suggestive of an abscess (Figure 1). His liver was enlarged with a span of 16.6 cm with no other focal lesion. No perihepatic or pleural effusion was seen. All other abdominal viscera appeared normal with no free fluid.

Routine hematology investigations showed hemoglobin 9.3 gm/dl; total leukocyte count (TLC) 22,600/mm^3^; differential leukocyte count (DLC): polymorphs 76, lymphocytes 20, monocytes 2, eosinophils 2; platelet count 3.76 lac; erythrocyte sedimentation rate (ESR) 52 mm at the end of the first hour; blood urea 28 mg%; prothrombin time of 15 seconds with a control of 10.1 seconds; total bilirubin 1 mg%; serum alkaline phosphatase 7 IU; serum glutamic oxaloacetic acid transaminase (SGOT) 29 IU; serum glutamic pyruvate transaminase (SGPT) 39 IU; serum protein 6.6 g/dl and serum albumin 4.0 g/dl.

Pigtail catheterization was carried out under local anesthetic (LA), which drained 150 ml of cream-colored pus. The pus was sent for Ziehl-Neelsen (ZN) staining, acid-fast bacilli (AFB) culture using a BACTEC TB 460 instrument, polymerase chain reaction (PCR) assay for *Mycobacterium tuberculosis *and other routine microbiological investigations (wet mount, Gram stain, pus culture for bacteria and fungi). ZN staining showed AFB and BACTEC culture confirmed the same by isolating *M. tuberculosis *(by para-nitro-alpha-acetylamino-beta-hydroxy-propiophenone (NAP) test). Antibiotic sensitivity was performed with the BACTEC TB 460 instrument and the isolate was sensitive to isoniazid, streptomycin, rifampicin and ethambutol. PCR assay of the aspirate was also positive for *M. tuberculosis *DNA. The lysozyme proteinase K extraction method was used to extract the DNA. The target for amplification was a MPB 64 gene which has a 240 bp sized amplicon. The primers used in the PCR assay were as follows:

Primer 1: 5'-TCC GCT GCC AGT CGT CTTCC-3'

Primer 2: 5'-GTC CTC GCG AGT CTA GGCCA-3'

Other routine microbiological investigations did not reveal any significant findings. Wet mount showed no trophozoites of *Entamoeba histolytica *or any fungal element. A routine bacteriological culture was sterile. Three consecutive early morning sputum samples were also screened for AFB and were negative. The patient was non-reactive in HIV serology. He was started on systemic antitubercular treatment (ATT) on receipt of the results from the laboratory, and this included isoniazid (300 mg once daily), rifampicin (450 mg once daily), pyrazinamide (1200 mg once daily), ethambutol (750 mg once daily) and pyridoxine (20 mg once daily). His fever subsided, and his appetite and general condition improved. He was discharged with stable vitals and asked to come for follow-up checkups after 6 weeks. At the first follow-up visit, the patient was asymptomatic, his liver size had decreased (size 3.1 × 3.8 × 5.1 cm) and a repeat USG abdomen revealed regression of the abscess.

## Discussion

In extrapulmonary tuberculosis, hepatic tuberculosis has been regarded as a rare form of TB [4]. Bristowe first described TLA in 1858 [5]. Most of the cases described usually occurred in association with miliary tuberculosis, mainly through hematogenous dissemination. The respiratory and GI tracts were the major sources of infection and bacilli travelled there via hepatic artery or the portal vein [6]. Levine classified hepatic tuberculosis into various forms of presentation such as (i) miliary tuberculosis, (ii) primary pulmonary tuberculosis with liver involvement, (iii) primary liver tuberculosis, (iv) tuberculoma, and (v) tuberculous cholangitis [7].

The prevalence of TLA was just 0.34% in patients with hepatic tuberculosis as shown in a study where the patient age ranged from 6 months to 72 years with an average age of 39.2 years [8]. Symptoms of the disease are commonly non-specific and include fever, vague abdominal pain, anorexia and weight loss [9]. Hepatomegaly is a common physical finding. Jaundice is a very rare manifestation of TLA and may be caused by extra- or intrahepatic obstruction. No clear relationship exists between the degree of liver involvement and jaundice [10]. TLA is frequently confused with hepatoma, pyogenic liver abscess and amoebic liver abscess. Because of the non-specific clinical presentation, the diagnosis of TLA is usually made at autopsy or occasionally after laparotomy has been performed [2].

Our patient was first treated for amoebic liver abscess. Amebiasis is endemic in India and antibodies may appear even in non-invasive infections of *Entamoeba histolytica *and may persist years after clinical cure. Amoebic serology reports may therefore prove to be misleading and much important therapeutic time may be lost unless a high index of clinical suspicion is maintained.

The radiological findings of TLA have a low specificity [11]. USG and computed tomography (CT) scan findings usually reflect different stages of disease varying from granulomatous tubercles with or without caseous necrosis to fibrosis and calcification in the healing stage [6]. USG findings of hepatic tuberculosis usually show hypo-echoic lesions [12], as also seen in our patient, but few studies have demonstrated hyperechoic lesions as well [4]. Therefore, the ultimate diagnosis of TLA depends upon the demonstration of AFB in pus, aspirate or biopsy specimen or the necrotic tissue.

AFB is most easily found in caseous necrotic material but even the absence of AFB should not detract from diagnosis, especially in a high TB prevalence country such as ours, as is evident from some other studies [4]. Recently, PCR has been found to be a useful diagnostic tool for hepatic tuberculosis [11] as it enables rapid identification of *Mycobacterium tuberculosis *and expedites a treatment decision. At least 57% of tuberculous hepatic granulomas gave positive PCR results compared to other conventional diagnostic techniques for TB [13]. Another advantage is that PCR analysis can distinguish *M. tuberculosis *from other mycobacterium saving a lot of precious time.

Medical treatment of tuberculous liver abscess is still a subject of debate. Gracey postulates that thick fibrous tissue around the abscesses and their large size may prevent antibiotics from reaching the target [14]. Quadruple therapy with antitubercular drugs is recommended for 1 year. Percutaneous drainage of the abscess, combined with systemic ATT has been used in appropriate cases [15]. In some other cases, TLAs have been successfully treated by percutaneous drainage combined with transcatheter infusion of antitubercular drugs [9,16]. In our case, the patient responded well to systemic ATT and was improving when last seen 6 weeks after starting ATT.

## Conclusion

This is a rare case of an isolated hepatic tubercular abscess without any pulmonary and GI tract foci in an immunocompetent adult. The clinical presentation of an isolated TLA is so atypical that it challenges the clinical acumen of the treating physician, and hence a high index of suspicion should be maintained when dealing with a space-occupying lesion in the liver so that evidence-based specific management may be undertaken instead of empirical therapy. The prognosis of a hepatic tubercular abscess is excellent for the majority of patients if diagnosed early and prompt treatment is administered.

## Abbreviations

AFB: acid fast bacilli; ALA: amoebic liver abscess; ATT: antitubercular treatment; CT: computed tomography; CVS: cardiovascular system; DLC: differential leukocyte count; DNA: deoxyribonucleic acid; ESR: erythrocyte sedimentation rate; GI: gastrointestinal; HIV: human immunodeficiency virus; LA: local anesthetic; NAP: para-nitro-alpha-acetylamino-beta-hydroxy-propiophenone; OPD: outpatient department; PCR: polymerase chain reaction; SGOT: serum glutamic oxaloacetic acid transaminase; SGPT: serum glutamic pyruvate transaminase; TB: tuberculosis; TLA: tuberculous liver abscess; TLC: total leukocyte count; USG: ultrasonogram; ZN: Ziehl-Neelsen

## Competing interests

The authors declare that they have no competing interests.

## Authors' contributions

CPB analyzed and interpreted the patient data regarding the presentation and uniqueness of the disease and provided guidance during the entire exercise of the patient work-up. VG performed the laboratory procedures detailed in the case report. MC was a major contributor in collecting literature information and writing the manuscript. HJ provided help and support in the laboratory analysis. All authors read and approved the final manuscript.

## Consent

Written informed consent was obtained from the patient for publication of this case report and any accompanying images. A copy of the written consent is available for review by the Editor-in-Chief of this journal.
